# Incidence, Disease Spectrum, and Outcomes of Tuberculous Meningitis in South African Children: The Initial Impact of COVID-19

**DOI:** 10.3390/tropicalmed10050127

**Published:** 2025-05-07

**Authors:** Victoria E. Namukuta, Mariette Smith, Danite Bester, Magriet van Niekerk, Regan Solomons, Ronald van Toorn, Hendrik Simon Schaaf, James A. Seddon, Helena Rabie, Mary-Ann Davies, Anneke C. Hesseling, Karen du Preez

**Affiliations:** 1 Division of Epidemiology and Biostatistics, Department of Global Health, Stellenbosch University, Cape Town 7505, South Africa; 2 Desmond Tutu TB Centre, Department of Paediatrics and Child Health, Stellenbosch University, Cape Town 8000, South Africahss@sun.ac.za (H.S.S.); jseddon@sun.ac.za (J.A.S.); annekeh@sun.ac.za (A.C.H.); karendp@sun.ac.za (K.d.P.); 3 Health Intelligence, Western Cape Government Health and Wellness, Cape Town 8000, South Africa; mariette.smith@westerncape.gov.za (M.S.); mary-ann.davies@uct.ac.za (M.-A.D.); 4 Center for Integrated Data and Epidemiological Research, School of Public Health, University of Cape Town, Cape Town 7701, South Africa; 5 Department of Paediatrics and Child Health, Faculty of Medicine and Health Sciences, Stellenbosch University, Cape Town 7505, South Africa; magrietvn@sun.ac.za (M.v.N.); regan@sun.ac.za (R.S.); vtoorn@sun.ac.za (R.v.T.); hrabie@sun.ac.za (H.R.); 6 Department of Infectious Disease, Imperial College London, London SW7 2AZ, UK

**Keywords:** tuberculous meningitis, COVID-19, SARS-CoV-2, paediatric, surveillance

## Abstract

Tuberculous meningitis (TBM) is a very severe form of childhood tuberculosis (TB), requiring hospitalisation for diagnosis. We investigated trends in admission, disease spectrum, outcomes, and healthcare system factors in children with TBM managed at a tertiary referral hospital in Cape Town, South Africa. We conducted a retrospective cohort study of children (<13 years) with TBM admitted from 2017 to 2021. An innovative surveillance algorithm was used to identify all possible TBM episodes using integrated electronic health data. Episodes were clinically verified and data were extracted using medical records. A total of 263 children (median age 2.2 years; IQR: 1.1–5.1), 17 (6.5%) living with HIV were admitted with TBM during 2017 to 2021. There was a significant reduction in TBM admissions during the COVID-19 pandemic (IRR: 0.57, 95% CI:0.39–0.84), particularly in children < 2 years (IRR: 0.31, 95% CI: 0.15–0.62). BCG vaccination was documented in 137/263 (52.1%) and 10/87 (11.5%) eligible children who initiated TB preventive therapy. During the pandemic, children with TBM were significantly more likely to be living with HIV (aOR: 4.01, 95% CI: 1.39–11.62). COVID-19 was associated with a significant reduction in the number of young children admitted with TBM. Many missed opportunities to prevent TBM were identified regardless of COVID-19. Paediatric TBM surveillance is a useful marker to monitor epidemiological trends.

## 1. Introduction

Tuberculous meningitis (TBM) in children is characterised by high levels of morbidity and mortality [1]. Following exposure to Mycobacterium tuberculosis (*M.tb*), young children, particularly those below 2 years of age and those living with HIV, are at high risk of tuberculosis (TB), including disseminated disease, of which TBM is the most serious form [2]. The early stages of TBM in young children are often characterised by nonspecific symptoms, resulting in missed or delayed diagnosis, and presentation at advanced clinical disease stage, which is associated with poor neurological outcomes and high mortality [1,3].

South Africa is classified by the World Health Organization (WHO) as one of the 30 high-TB-burden countries globally, with an estimated 23,100 children aged <15 years developing TB in 2023. TB in children contributes 8.5% of the overall national TB burden [4]. In the Western Cape Province of South Africa, TBM was identified as the leading cause of bacterial meningitis, with the proportion of children with TBM of those overall treated for meningitis having more than doubled over the two decades between 1986 and 2009 at a tertiary hospital [5,6]. Between 1985 and 1993, 282 of 2920 (9.7%) children with meningitis had TBM, while from 2007 to 2009, 122 of 557 (22%) children with meningitis had TBM [5,6]. Given the need for specialized imaging and clinical care of TBM, it is highly likely that children with TBM will eventually be admitted and diagnosed at a hospital for care. Without treatment, death is almost always inevitable with TBM [7].

Nearly 20% (77/395) of the total paediatric TB case load at Tygerberg Hospital (TBH), a large tertiary referral hospital in the Western Cape serving approximately half of the provincial paediatric population, was disseminated TB, including TBM and miliary TB in 2012 [8]. Ongoing surveillance for TBM at this hospital has highlighted the impact of bacille Calmette–Guérin (BCG) vaccine stockouts in 2015 on the number of children treated for TBM, with paediatric TBM or intracranial tuberculoma admissions increasing more than twofold in 2017 following the 2015 vaccine stockouts [9].

Incident TB and TBM in young children reflect recent *M.tb* transmission and therefore are potential indicators of TB control and health system functioning in a given setting [10,11]. Surveillance of TBM in children is also a valuable tool to measure and respond to modifiable risk factors such as BCG vaccination, TB contact management, tuberculosis preventive treatment (TPT), and the impact of COVID-19 [10]. Since early 2020, the COVID-19 pandemic devastated health systems globally with a detrimental effect on TB preventive, diagnostic, and treatment services and the first reversal of a steady decline observed in TB mortality in 10 years [12,13]. Globally, there was a significant decline in paediatric TB notifications, especially in young children [14].

In South Africa, the first and strictest lockdown was instituted on 26 March 2020, shortly after the WHO declared COVID-19 a pandemic, and this lasted 35 days [15]. The country cycled through five Alert levels, with level five being the strictest measure restricting public movement and gatherings, with the aim to prepare existing health systems to manage COVID-19 cases and curb the rate of transmission of the SARS-CoV-2 virus [16]. The level five lockdown was stepped down to Alert level four after 35 days, followed by Alert level three after 31 days, Alert level two after 47 days, and, finally, Alert level one on 21 September 2020 until 28 December 2020 [16]. In 2021, the country cycled through Alert levels one–to-four with levels three and four instituted between 16 June 2021 and 12 September 2021 [16].

For each Alert level, hand sanitizing, social distancing, and wearing of face masks were maintained. As resources and facilities were repurposed for management of the anticipated COVID-19 case surge, hospital admissions and routine primary healthcare services were restricted, and any children who could be treated at home were managed on an outpatient basis [17]. Between April and July 2020, early reports estimated that 308,000 fewer Xpert MTB/RIF Ultra tests were conducted during the level five and level four lockdowns [18]. In the Western Cape, a study evaluating the effect of the pandemic on the number of people diagnosed with and treated for drug-susceptible (DS)-TB found a 28% relative reduction in diagnosis, and a significant increase in pre- and post-treatment loss to follow-up during the pandemic [19].

Although several studies have assessed the effect of the COVID-19 pandemic on TB generally, none have specifically evaluated the impact on the incidence and spectrum of TBM in children. As all children with TBM require hospital admission, hospital surveillance of these admissions can serve as a sensitive marker of paediatric TBM, thereby providing insight into the impact of COVID-19 on TB epidemiology more broadly. Despite the restructuring of public healthcare systems to better manage the COVID-19 pandemic, children with TBM would still have been classified as medical emergencies and managed as inpatients. We aimed to characterize and compare the burden, disease spectrum, clinical characteristics, and associated healthcare system factors in children with TBM managed at a tertiary referral hospital in Cape Town, South Africa, before and during the COVID-19 pandemic period.

## 2. Materials and Methods

### 2.1. Study Design, Population and Setting

This was a retrospective cohort study including all children (<13 years of age) routinely admitted with or treated for TBM at TBH, a tertiary referral hospital in Cape Town, South Africa, over a 5-year period (1 January 2017 to 31 December 2021). Children aged <13 years were classified as paediatric patients in this public hospital setting. TBH serves as one of two tertiary-level hospitals for the Western Cape province with a population of 7.4 million people, of whom 22.4% are children 0–14 years old [20].

### 2.2. Case Identification

In collaboration with the Provincial Health Data Centre (PHDC), we supported the development of a novel surveillance algorithm to identify possible TBM episodes using integrated electronic data from the PHDC. The PHDC is exclusively housed within the Western Cape Province Department of Health. It is a comprehensive health information exchange system that uses a unique patient identifier to consolidate individual clinical data across public health services, including, but not limited to, data from hospitals, primary healthcare facilities, pharmacies, and laboratories in the province. As most patients utilise public healthcare facilities for TB and HIV services, the PHDC is a reliable source of epidemiological data for these conditions [21]. The algorithm used to identify TBM episodes integrated biochemistry, microbiological, clinical (ICD10 coding), and pharmacy data to identify children with potential TBM.

All possible TBM episodes were independently assessed against source medical folders to determine the final diagnosis by an experienced study nurse. Any queries and discrepancies were reviewed and resolved by the principal investigator and a senior paediatrician.

### 2.3. Data Collection and Definitions

Clinical patient-level data were collected through retrospective folder reviews from hospital electronic medical records. Key variables included age, sex, weight, the basis of TBM diagnosis and clinical staging, BCG vaccination status, TB exposure history, TPT status, HIV exposure and infection status, microbiological investigations, radiological investigations and findings, in-hospital, and final TBM treatment outcomes. Using WHO 2007 growth reference standards, we calculated weight-for-age z-scores for children 0 to 10 years whose weights were recorded [22,23,24]. The WHO weight-for-age reference standards are limited to children under 10 years [24]. Children with weight-for-age z-score of <−3 standard deviations (SDs) from the expected average z-score for age were classified as severely underweight. Weight-for-age z-scores ≥−3 and <−2 SD were classified as underweight. Weight-for-age z-scores ≥−2 were classified as normal weight.

Clinical staging in children with TBM is routinely determined according to the British Medical Research Council (BMRC) severity grade. It includes three stages: Stage 1 (Glasgow Coma Score [GCS] score 15; no focal neurological signs), Stage 2 (GCS score 10–14 with or without focal neurological signs, or 15 with focal neurological signs) and Stage 3 (GCS score < 10). Stage 2 is further divided into stages 2a (GCS score 13–14, or 15 with focal neurological signs) and 2b (GCS 10–12, with or without focal neurological signs) [25]. We used the recorded clinical stage or calculated it from the available information in the folders.

TB exposure was defined as close contact with an infectious pulmonary TB source patient living in the same household as, or who was in frequent contact with the child within the past 12 months [26], documented in the medical records. According to TPT guidelines during the study period, children < 5 years and children ≥ 5 years living with HIV (CLWH) were eligible to receive TPT [26]. BCG vaccination was considered to have been given if medical records indicated that “BCG vaccination status was up to date” or “Immunizations up to date” or if there was a BCG scar present on clinical examination.

TBM treatment outcomes were assessed and verified using all available recorded clinical information. According to the 2013 South African paediatric TB treatment guidelines, the duration of routine treatment for DS-TBM is six months for children who are not living with HIV and nine months for CLWH. Children with confirmed or presumed DS-TBM are treated with a four-drug regimen that includes daily isoniazid (20 mg/kg), rifampicin (20 mg/kg), pyrazinamide (40 mg/kg), and ethionamide (20 mg/kg) for the full duration of treatment. Drug-resistant forms of disease are treated with individualised regimens. For children whose treatment outcome was not recorded or missing from clinical records, we assumed TB treatment completion if we found any documented evidence that they had received their last month of treatment. Favourable treatment outcomes were defined as cure or treatment completion, whereas unfavourable outcomes were defined as death, loss to follow-up, treatment failure, or unknown treatment outcomes. Early in-hospital outcome refers to whether the child died in hospital or if they were discharged from hospital.

### 2.4. Statistical Analysis

De-identified data were analysed using STATA BE 18.5 (Stata Corp LLC, College Station, TX, USA). Summary continuous data were reported as median with interquartile range (IQR) and mean with standard deviation (SD). Categorical data were reported as frequencies and percentages. For comparison of demographic and clinical characteristics pre-COVID-19 vs. during COVID-19 pandemic periods, 1 April was used as the cut-off date dividing the two cohorts because this approximates the date when South Africa declared her first total lockdown (26 March 2020). Demographic and clinical characteristics were compared before and during the COVID-19 pandemic using the Mann–Whitney U-test for continuous data and Pearson chi square test (Fisher’s exact test if assumptions not met) for categorical data.

We graphically reported the annual trends in the number of paediatric TBM episodes at TBH by calendar year starting 1 January and ending 31 December for each year. A Poisson regression was used to estimate an incidence rate ratio (IRR) with 95% confidence intervals (CI) to compare the average annual number of admissions before (1 April 2017 to 31 March 2020) and during (1 April 2020 to 31 March 2021) the COVID-19 pandemic periods. There were no formal changes in referral pathways during the study period and we assumed that the population catchment area of TBH remained constant throughout the study period.

We also compared the average annual number of children age < 13 years at TBH with microbiologically confirmed TB (Xpert MTB/RIF Ultra and/or culture) before and during the COVID-19 pandemic using a Poisson regression. For this analysis, we used the same time period as above (unpublished data, surveillance study personal communication Prof. Schaaf).

Univariable and multivariable analyses were completed to compare the clinical characteristics, healthcare system factors and outcomes before and during the COVID-19 pandemic periods with 1 April 2020 as the cut-off date dividing the two cohorts. Age, sex, HIV exposure, and infection status were included as a priori variables in the multivariable model, as well as variables that were significant at alpha ≤ 0.05 in univariable analysis. The Hosmer–Lemeshow test was used to assess the final multivariable model for goodness of fit.

Kaplan–Meier survival probability was plotted for children with DS-TB with known treatment outcomes (death or completed treatment) to compare favourable vs. unfavourable outcomes before and during the COVID-19 pandemic. Duration of follow-up was censored at 10 months because both HIV-negative children and CLWH would have completed treatment according to local guidelines for treatment of TBM. Comparison of survival curves was performed using the log-rank test.

### 2.5. Regulatory Approval

Ethics approval for this study was obtained from the Stellenbosch University Health Research and Ethics Committee (S23/11/279) and regulatory approvals from TBH (Project ID 22167) and the Western Cape Department of Health (WC_202107_021). Because the research was limited to retrospective routine medical record reviews, a waiver of individual informed consent was obtained.

## 3. Results

### 3.1. Demographic Characteristics

The algorithm identified 392 possible TBM episodes, of which 263 were clinically verified as having TBM between 2017 and 2021 (Table 1). The median age of children was 2.22 years (IQR:1.13–5.14). Median age of children prior to the COVID-19 pandemic was 2.02 years (IQR: 1.05–4.70), significantly lower compared to the pandemic period (3.14 years, IQR: 1.82–6.23). Of 212, 51 (24.1%) children were severely underweight.

### 3.2. Trends in TBM Episodes

**Figure 1:**
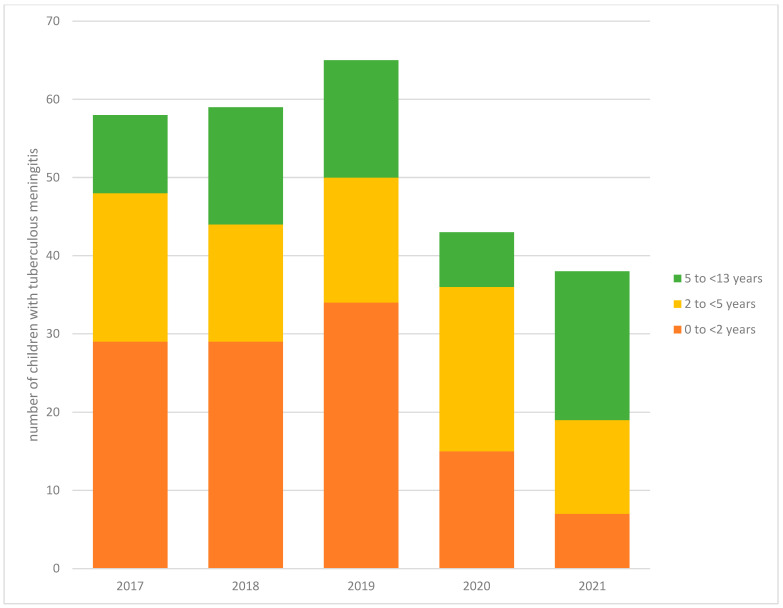
Trends in the number of children with tuberculous meningitis at Tygerberg Hospital from 2017 to 2021 stratified by age (N = 263).

There was a significant reduction in the number of TBM episodes managed at TBH during the COVID-19 pandemic (mean = 35) compared to the average annual number of admissions for the preceding 2 years (mean = 62) (IRR: 0.57, 95% CI: 0.39–0.84) (Table 2, Figure 1). When the IRR was stratified by age category, the association only remained significant for the youngest children (0–2 years of age; IRR: 0.31, 95% CI: 0.15–0.62). More generally, there was a 50% reduction in microbiologically confirmed TB in 0- to 13-year-old children at TBH during the COVID-19 pandemic (mean = 130) compared to before the pandemic (mean = 248, IRR: 0.50, 95% CI: 0.49–0.60, *p*-value < 0.001).

### 3.3. History and Clinical Characteristics

Of 263 children, 79 (30.0%) presented with stage 3 TBM (Table 1). Of 263 children, 17 (6.5%) were treated for DR-TBM. Of these, 3/17 (17.6%) children were treated for isoniazid mono-resistant TBM, 4/17 (23.5%) children were treated for rifampicin-resistant TBM, and 10/17 (58.8%) children were treated for multidrug-resistant (MDR)-TBM.

Only 179/263 (68.1%) children had any data recorded about BCG vaccination status, with only 137/179 (76.5%) having documented evidence of BCG vaccination, and a further 84/263 (31.9%) children with BCG vaccination status unknown or not recorded (Table 1). A recent TB exposure history was documented in 100/263 (38.0%) children. Of the 100 children who reported TB exposure, we could not ascertain TPT eligibility for one child because they were >5 years old and their HIV status was unknown. Of 99 children, 87 (87.9%) were eligible to receive TPT according to local guidelines. TPT initiation, however, was only documented in 10/87 (11.5%) children. Of the children who reported TB exposure, 10/87 (11.5%) were treated for drug-resistant (DR)-TBM. TPT initiation was documented in only 2/10 (20%) children with DR-TBM with a history of TB exposure.

Of the 17 (6.5%) CLWH, 6/17 (37.5%) were diagnosed with HIV at the time of TB investigation, 5/17 (31.2%) had been on antiretroviral treatment (ART) prior to their TBM diagnosis, and 5/17 (31.2%) were known to be CLWH but were not currently on ART at the time of their TBM diagnosis.

Overall, 165 (62.7%) had microbiologically confirmed TB by either Xpert MTB/RIF and/or *M.tb* culture of CSF and/or other non-CSF specimen; 89/263 (33.8%) children were Xpert MTB/RIF positive (including trace positive) and/or *M.tb* culture positive on a CSF specimen, and an additional 76/263 (28.9%) had microbiologically confirmed TB on a non-CSF specimen (see Supplementary Material, Tables S1–S4 for disaggregated results).

### 3.4. Early (In-Hospital) and Final TB Treatment Outcomes

Thirteen (4.9%) children died in hospital (Table 3).

Of those who were discharged from TBH, more children continued receiving treatment at home with monthly hospital follow-up during the COVID-19 pandemic (61.9%) compared to the time period before (49.7%). Overall, 30/263 (11.5%) children died in hospital or post-discharge, 204/263 (77.6%) completed their treatment successfully, 27/263 (10.3%) children were lost to follow-up, and only 2/263 (0.8%) children had treatment failure.

### 3.5. Univariable and Multivariable Analysis

Children with TBM managed during the COVID-19 period were more likely to be older than 2 years (children 2 to <5 years old, OR: 2.80; 95% CI: 1.38–5.64; and children 5 to 13 years old, OR: 3.28; 95% CI: 1.58–6.81) and living with HIV (OR: 5.19; 95% CI, 1.92–14.80) than children before the COVID-19 pandemic (Table 4). Univariable analyses showed no significant differences in sex, TBM disease stage, nutritional status, BCG vaccination status, TPT initiation, in-hospital mortality, or overall treatment outcomes between the two time periods.

In the multivariable model adjusting for age, sex, and HIV status, the associations between the COVID-19 period and age and HIV status of children with TBM remained. During the COVID-19 period, children were approximately twice as likely to be older than 2 years (children 2- < 5-years old: aOR: 2.62 (95% CI: 1.28–5.39) and children 5–13-years old: aOR: 2.91, 95% CI: 1.35–6.28)) and four times more likely to be living with HIV (aOR: 4.01, 95% CI: 1.39–11.62). Sensitivity analysis was performed to assess whether there was a difference in treatment outcomes for children who were diagnosed in the “before COVID-19 period” but completed their TBM treatment during the COVID-19 pandemic compared to those who were diagnosed and completed treatment before the pandemic, and we found no statistically significant difference.

### 3.6. Survival Analysis

**Figure 2:**
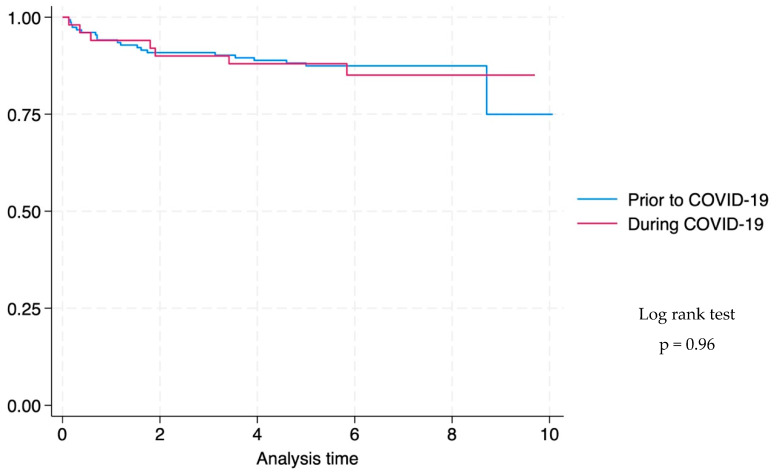
Kaplan–Meier survival graph comparing time to death of children with drug-susceptible tuberculous meningitis at Tygerberg Hospital prior to and during the COVID-19 pandemic ^a^ (n = 203). ^a^ Survival curves are only plotted for children with DS-TB with known treatment outcomes (death or completed treatment), and the duration of follow-up was censored at 10 months because both HIV-negative and HIV-positive children would have completed treatment according to local guidelines for treatment of TBM. Duration of treatment is six months for HIV-negative children and 9 months for children living with HIV.

There was no difference in survival time prior to and during the COVID-19 pandemic (log rank test *p*-value: 0.96) (Figure 2).

## 4. Discussion

This study showed a significant reduction in the total number of TBM episodes managed at a large provincial referral hospital in South Africa during the COVID-19 pandemic relative to the previous time period. This was largely driven by a decrease in the number of children below 2 years of age with TBM. Although the numbers were small, a greater proportion of TBM episodes were in children living with HIV (vs. without HIV) during the COVID-19 pandemic vs. the prior time period. We did not find any difference in BCG vaccination status, TPT initiation, TBM disease severity, or TBM treatment outcomes between the two periods, but we found many missed opportunities for prevention of TBM overall.

The decline of 43% in the total number of paediatric TBM episodes at TBH during the COVID-19 pandemic was substantial compared to the pre-pandemic annual average. In age-stratified analyses, we found that this difference was largely driven by a 69% decline of TBM in children under 2 years of age. This association with age remained significant in multivariable analysis adjusting for the effect of sex and HIV status. This is similar to findings from a modelling study that reported a significant decline in global paediatric TB notifications during the COVID-19 pandemic, with the greatest change seen in young children (0–4 years) [14]. Another study conducted early in the COVID-19 pandemic in South Africa also reported a decline in a paediatric TB diagnoses [27].

Within this context, there was a general reduction in paediatric admissions during the pandemic, most marked in the strictest (level five and level four) lockdown periods where public movement was severely limited and routine nonemergency primary healthcare visits indefinitely postponed to contain the spread of SARS-CoV-2 and increase hospital capacity to manage COVID-19 cases [28]. Although paediatric admissions declined substantially during the pandemic, we did not expect to see this change in children diagnosed with TBM given the disease pathogenesis and diagnostic and treatment pathways for TBM. One hypothesis is that these children were undiagnosed and died prior to accessing care during the pandemic. A study conducted at TBH found a significant increase in in-hospital mortality of children under 5 years during the pandemic [28]. However, because the study was limited to in-hospital mortality, it is unknown how the pandemic affected mortality at the community level. In the same study, there was a 1.8-fold decline in primary healthcare visits during level five and level four lockdowns in the Western Cape province of South Africa [28]. If the observed reduction in our study was due to undiagnosed deaths during the pandemic, the absolute numbers are likely relatively small and might not reflect in crude indicators such as under-5-mortality or infectious diseases deaths [29].

Conversely, there might have been a true reduction in TBM cases due to reduced TB transmission, as TB disease in children is an indicator of recent community TB transmission [10,11]. As *M.tb* is airborne and shares similar transmission patterns with SARS-CoV-2, measures such as social distancing, limited public gathering, and masking might have reduced *M.tb* exposure and transmission outside the home [30]. A study evaluating the impact of the pandemic on under-5 lower respiratory tract infection (LRTI) admissions and mortality in the Western Cape found that public health restrictions may have affected disease transmission patterns with subsequent reduction in admission rates [29]. Notably, our study design cannot ascertain whether there was reduced community transmission of *M.tb* or missed TBM diagnoses; therefore, untangling these effects and interpreting these findings is complex. It is worth noting that the BCG vaccine stockouts in 2015 might have increased the number of children under 2 years with TBM in the pre-COVID years, confounding the observed impact of COVID-19 on TBM in this age group. Further research is needed to fully understand the relative impact of the COVID-19 pandemic on TB transmission patterns, healthcare systems, and access, as well as changes in BCG vaccine coverage.

We observed that during the pandemic, children with TBM were significantly more likely to be living with HIV than in the pre-pandemic period. Although there was no statistically significant difference in timing of HIV diagnosis between the two cohorts, more children were lost to follow-up from their routine antiretroviral therapy (ART) during the pandemic. In South Africa, there was a significant reduction in HIV testing and treatment services at primary healthcare (PHC) level during the pandemic, especially among older children (5–14 years) [31,32]. Because untreated HIV infection is a significant risk factor for TBM disease in children [2], this could explain why there were more children with TBM and HIV during the COVID-19 pandemic. However, ART clinics did not close during the pandemic, and many strategies were employed to ensure that children did not run out of ART [33]. These findings highlight the need for sustained follow-up and retention in ART care among children living with HIV.

Despite its known efficacy, BCG vaccination was suboptimal across the entire study period. During the study period, BCG coverage in South Africa ranged from 70 to 86% [34], despite being routinely recommended to be given at birth to all infants. In our study, BCG vaccination status was not documented in one-third of children with TBM (32%), and of children with documented BCG vaccination status, only 76.5% received their BCG vaccine. BCG vaccination is safe and effective in protecting young children against TBM [35,36]. A systematic review found that the pandemic was associated with a 10% decline in paediatric vaccinations in low- and middle-income countries (LMICs), with the greatest change in vaccines given at birth [37]. A previous study showed that global shortages of the BCG vaccine during 2015 were closely associated with an increase in the number of paediatric TBM admissions in 2017 for children born during the local stock-out period at a tertiary hospital in Cape Town, South Africa [9]. These findings highlight the continued need for BCG vaccination in infants at the highest risk of TB and progression to TBM disease.

TPT is highly effective in reducing the risk of TB disease in children following exposure, yet TPT uptake remains challenging in high-TB-burden countries, including South Africa [38,39]. Our study found documentation of TPT initiation in very few (11.5%) eligible children. A course of TPT may have prevented TBM in some of these children, emphasizing the need for sustained initiatives to improve contact tracing, screening, and TPT initiation.

Despite the high TB burden and resource limitations in our setting, the observed mortality of 30/263 (11.5%) was low compared to a systematic review and meta-analysis of 1636 children that estimated mortality at 20%. Less than half of the observed deaths in our study, 13 (43.3%%), occurred in hospital, whilst 17 (56.7%) occurred after discharge [40]. Overall, favourable outcomes were recorded in 77.9% of children in our study, similar to those previously reported in the same setting (80%) [41].

From the onset of symptoms until treatment completion and rehabilitation, children with TBM navigate multiple complex levels of care that often result in diagnostic and treatment delays, in addition to catastrophic costs for affected families [42]. In this setting, children with TBM either complete treatment under a home-based care program or at a TB-specialised hospital depending on detailed social and clinical assessments by a social worker and the attending doctors. In our study, we found that significantly more children were discharged to home-based care during the pandemic, likely because TB-specialised hospitals were used as COVID-19 treatment facilities, reducing their capacity to manage routine TB treatment for socially challenged children and/or those with MDR-TB. From our study, this did not seem to have an impact on overall treatment outcomes during the pandemic, supporting previous findings that home-based care for TBM is safe, effective, and cost-friendly for children in this setting [41,43,44].

To the best of our knowledge, this study is the first to specifically evaluate the effect of the COVID-19 pandemic on the burden, spectrum, and outcomes of paediatric TBM. This study used an innovative surveillance strategy, employing an algorithm developed to identify all possible children with TBM using multiple integrated sources of electronic health data. This algorithm can be scaled for future surveillance studies of TBM in children and adults.

This study has some limitations. First, incomplete clinical records hampered our ability to ascertain clinical characteristics and treatment outcomes for some of the children with TBM. Every effort was made to trace the missing information using multiple available sources. Second, the study was not designed to measure the underlying causes of our observations, and future research is needed to investigate the effect of the pandemic on TB transmission and key epidemiological TB indicators. Postmortem data would be useful to assess whether TBM contributed to deaths in children who died prior to accessing healthcare. A final limitation is that the study was conducted between 2017 and 2021. Our results can only partially account for the influence of the COVID-19 pandemic on TBM trends, with longer-term surveillance studies encompassing the entire post-COVID-19 period (2022 and onwards) needed to gain a fuller understanding of how the pandemic altered paediatric TBM trends and how these were changed.

## 5. Conclusions

The COVID-19 pandemic was associated with a reduction in the number of children with TBM at a large academic hospital in South Africa, especially amongst children below 2 years of age. Children with TBM managed during the pandemic were nearly four times more likely to be living with HIV. BCG and TPT coverage were poor overall, regardless of COVID-19. Strengthening of healthcare services is required to improve the prevention of TBM in children. Surveillance of TBM in children is a useful tool to monitor epidemiological trends in the overall TB epidemic.

## Figures and Tables

**Table 1:** Demographic and clinical characteristics of children diagnosed with tuberculous meningitis at Tygerberg Hospital during vs. before the COVID-19 pandemic periods in Cape Town, South Africa (n = 263).

	Prior to COVID-19 Pandemic ^b^	During COVID-19 Pandemic ^c^	Total	*p*-Value ^a^
	n (%)	n (%)	N (%)	
**Number (%)**	198 (75.3)	65 (24.7)	263 (100.0)	
**Age bands**				
0 to <2 y	98 (49.5)	16 (24.6)	114 (43.3)	0.002
2 to <5 y	57 (28.8)	26 (40.0)	83 (31.6)	
5 to <13 y	43 (21.7)	23 (35.4)	66 (25.1)	
**Sex**				
Male	96 (48.7)	40 (60.6)	136 (51.7)	0.095
Female	101 (51.3)	26 (39.4)	127 (48.3)	
**Nutrition status (weight for age z-score) ^d^**				
**Severely underweight**	38 (24.2)	13 (23.6)	51 (24.1)	0.978
**Underweight**	33 (21.0)	11 (20.0)	44 (20.8)	
**Normal weight**	86 (54.8)	31 (56.4)	117 (55.2)	
**BCG vaccinated**				
Yes	104 (52.5)	33 (50.8)	137 (52.1)	0.267
No	35 (17.7)	7 (10.8)	42 (16.0)	
Vaccination status recorded as unknown/not recorded	59 (29.8)	25 (38.5)	84 (31.9)	
**TB contact history in the last 12 months**				
Yes	79 (39.9)	21 (32.3)	100 (38.0)	0.307
No	98 (49.5)	33 (50.8)	131 (49.8)	
No data on contact history	21 (10.6)	11 (16.9)	32 (12.2)	
**Eligible for TPT ^e^ (n = 99)**				
Yes	68 (87.2)	19 (90.5)	87 (87.9)	0.681
No	10(12.8)	2(9.5)	12 (12.1)	
**TPT initiated ^f^ (n = 87)**				
Yes	9 (13.2)	1 (5.3)	10 (11.5)	0.147
No	26 (38.2)	4 (21.1)	30 (34.5)	
**Unknown**	33 (48.5)	14 (73.7)	47 (54.0)	
**TBM stage at admission**				
Stage 1	39 (19.7)	15 (23.1)	54 (20.5)	0.832
Stage 2a	50 (25.3)	16 (24.6)	66 (25.1)	
Stage 2b	41 (20.7)	13 (20.0)	54 (20.5)	
Stage 3	59 (29.8)	20 (30.8)	79 (30.0)	
Not recorded	9 (4.5)	1 (1.5)	10 (3.8)	
**HIV status**				
Positive	7 (3.5)	10 (15.4)	17 (6.5)	0.001
Exposed uninfected	18 (9.1)	9 (13.8)	27 (10.3)	
Negative	168 (84.8)	45 (69.2)	213 (81.0)	
HIV status unknown	5 (2.0)	1 (1.5)	6 (2.3)	
**If living with HIV ^g^ (n = 16)**				
Newly diagnosed	2 (33.3)	4 (40.0)	6 (37.5)	0.411
On ART preceding TBM diagnosis	3 (50.0)	2 (20.0)	5 (31.2)	
Not on ART at time of TBM diagnosis	1 (16.7)	4 (40.0)	5 (31.2)	
**CSF Xpert (n = 192) ^h^**				
Positive	40 (28.8)	14 (26.4)	54 (28.1)	0.510
Trace positive ^i^	15 (10.8)	9 (17.0)	24 (12.5)	
Negative	84 (60.4)	30 (56.6)	114 (59.4)	
**CSF culture (n = 77) ^j^**				
Positive	13 (22.0)	5 (27.8)	18 (23.4)	0.614
Negative	46 (78.0)	13 (72.2)	59 (76.6)	
**Microbiological confirmation by CSF or another specimen ^k^**				
Xpert Ultra and/or culture positive	128 (64.6)	37 (56.9)	165 (62.7)	0.520
Xpert Ultra and/or culture negative	64 (32.3)	26 (40.0)	90 (34.2)	
Xpert Ultra and culture missing	6 (3.0)	2 (3.1)	8 (3.0)	
**Treatment received**				
Drug-susceptible TBM	185 (93.4)	61 (93.8)	246 (93.5)	0.907
Drug-resistant TBM ^l^	13 (6.6)	4 (6.2)	17 (6.5)	

^a^ *p*-values compared number of children with TBM for each outcome variable during vs. before the COVID-19 pandemic. Clinical characteristics were compared using the Mann–Whitney U-test for continuous data (age) and Pearson’s chi-square test (Fisher’s exact test if assumptions not met) for categorical data. ^b^ 1 January 2017 to 31 March 2020. ^c^ 1 April 2020 to 31 December 2021. ^d^ Weight-for-age z-scores were only calculated for children aged 0 to 10 years according to WHO 2007 growth reference standards. Children with weight-for-age z-score of <−3 standard deviations (SD) from the expected average z-score for age were classified as severely underweight, those with weight-for-age z-scores between −3 and −2 SD were classified as underweight, and those with weight for age z-scores >−2 were classified as normal weight. Weight was missing for 9 children. Twenty-four children >10 years were excluded from the weight-for-age z-score calculation and analysis. For 18 children, the weight for age z-scores were missing because they were more than 5 standard deviations from the mean z-score. ^e^ This outcome aimed to assess the number of children with known TB contact history in the previous 12 months who would have been eligible for TPT. Of 100 children that reported known recent TB exposure in the 12 months prior to TBM diagnosis, we could not determine TPT eligibility for one child because they were >5 years old and the HIV status was unknown. ^f^ According to local guidelines, 87/100 children with documented TB exposure were eligible to receive TPT because of their age or HIV status. ^g^ For one child, the clinical records did not indicate whether they were newly diagnosed, previously on ART, or if they had been lost-to-follow-up from routine HIV care. ^h^ Seventy-one children had unknown or missing CSF Xpert MTB/RIF results. ^i^ Twenty-four children with CSF Xpert MTB/RIF trace positive results were classified as bacteriologically confirmed tuberculous meningitis. Of these, 3 children had a positive CSF *M.tb* culture, 7 had a negative CSF *M.tb* culture, and 14 had missing or unknown CSF *M.tb* culture results. ^j^ One-hundred-eighty-six children had unknown or missing CSF culture results. ^k^ Of 165 children who had a positive Xpert MTB/RIF and/or culture on CSF or other specimen, 50 children had a positive Xpert Ultra and/or *M.tb* culture result for both CSF and other specimen, 39 children had only Xpert MTB/RIF and/or *M.tb* culture positive results on CSF, and 76 children had only Xpert MTB/RIF and/or *M.tb* culture positive result on specimens other than CSF. CSF culture for *M.tb* was not performed or unknown for 186 children. ^l^ Three isoniazid mono-resistant TB, 4 rifampicin-resistant TBM and 10 multidrug-resistant TBM. IQR: interquartile range, SD: standard deviation, y: years, BCG: *bacille Calmette–Guérin*, GCS: Glasgow Coma Scale, CSF: cerebrospinal fluid, *M.tb: Mycobacterium tuberculosis*, TPT: TB preventive treatment, HIV: human immunodeficiency virus.

**Table 2:** Incidence rate ratio comparing the number of children with tuberculous meningitis at Tygerberg Hospital prior to and during the COVID-19 pandemic, stratified by age band (N = 263).

	IRR ^a^	95% CI	*p*-Value
**Overall**	0.57	0.39–0.84	0.004
**Age stratified**			
0 to <2 years	0.31	0.15–0.62	0.001
2 to <5 years	0.92	0.51–1.67	0.793
5 to <13 years	0.70	0.34–1.44	0.333

^a^ For comparison, each calendar year began on 1 April and ended on 31 March. Therefore, we compared the number of TBM episodes for April 2018–March 2019 and April 2019–March 2020 with April 2020–March 2021. This is because the first lockdown in South Africa was instituted on 26 March 2020. The average annual number of admissions for the pre-pandemic period was 62, while the average annual number of admissions during the COVID-19 pandemic period was 35.

**Table 3:** Early in-hospital and final treatment outcomes of children with tuberculous meningitis at Tygerberg hospital prior to and during the COVID-19 pandemic (n = 263).

	Prior to COVID-19 Pandemic	During COVID-19 Pandemic	Total	*p*-Value ^c^
	n (%)	n (%)	N (%)	
**Number (%)**	198 (75.3)	65 (24.7)	263 (100)	
**Outcome at hospital discharge (n = 263)**				
Alive	187 (94.4)	63 (96.9)	250 (95.1)	0.424
Died	11 (5.6)	2 (3.1)	13 (4.9)	
**Healthcare facility referred to after discharge (n = 250)**				
Home-based care	93 (49.7)	39 (61.9)	132 (52.8)	0.002 ^a^
Medium-term facility	0 (0.0)	1 (1.6)	1 (0.4)	
TB hospital	88 (47.1)	16 (25.4)	104 (41.6)	
Other	6 (3.2)	6 (9.5)	12 (4.8)	
Not recorded	0 (0.0)	1 (1.6)	1 (0.4)	
**TBM Treatment final outcomes ^a^ (n = 263)**				
Cured/Treatment completed	155 (78.3)	49 (75.4)	204 (77.6)	0.772
Lost to follow-up	19 (9.6)	8 (12.3)	27 (10.3)	
Died	22 (11.1)	8 (12.3)	30 (11.4)	
Treatment failure	2 (1.0)	0 (0.0)	2 (0.8)	
**Binary treatment outcomes ^b^ (n = 263)**				
Favourable	155 (78.3)	49 (75.4)	204 (77.6)	0.627
Unfavourable	43 (21.7)	16 (24.6)	59 (22.4)	

^a^ Children with unrecorded treatment completion in source documents were classified as having completed treatment if they were HIV-negative and followed up for at least 5 months or HIV-positive and followed up for at least 8 months since the date of TBM diagnosis. We assumed that all children started TBM treatment on the date of TBM diagnosis. Children lost to follow-up were those for whom the treatment duration could not be verified or was less than recommended according to local treatment guidelines for TBM. ^b^ Favourable outcomes referred to cure or treatment completion whereas unfavourable outcomes referred to death, loss to follow-up, or treatment failure. ^c^ Clinical characteristics were compared using the Mann–Whitney U-test for continuous data (age) and Pearson’s chi-square test (Fisher’s exact test if assumptions not met) for categorical data. TBM: tuberculous meningitis.

**Table 4:** Univariable and multivariable analysis of clinical characteristics, healthcare system factors, and outcomes associated with children having tuberculous meningitis at Tygerberg hospital during the COVID-19 pandemic vs. before (n = 263).

Predictor Variable	OR ^a^	95% CI	*p*-Value	aOR	95% CI	*p*-Value
**Age band (n = 263)**						
0 to <2 years	Reference					
2 to <5 years	2.80	1.38–5.64	0.004	2.62	1.28–5.39	0.009
5 to <13 years	3.28	1.58–6.81	0.001	2.91	1.35–6.28	0.006
**Sex (n = 263)**						
Male						
Female	0.64	0.36–1.13	0.125	0.74	0.40–1.35	0.323
**HIV (n = 257)**						
Negative	Reference					
Exposed uninfected	1.87	0.79–4.43	0.157	1.91	0.79–4.63	0.152
Positive	5.19	1.92–14.80	0.001	4.01	1.39–11.62	0.010
**BCG vaccination (n = 179)**						
No	Reference					
Yes	1.59	0.64–3.91	0.315	–	–	–
**TPT initiation (n = 40)**				–	–	–
No	Reference					
Yes	0.72	0.07–7.34	0.783	–	–	–
**TBM staging (n = 253)**				–	–	–
Stage 1	Reference					
Stage 2a	0.83	0.37–1.89	0.660			
Stage 2b	0.82	0.35–1.95	0.661			
Stage 3	0.88	0.40–1.93	0.752			
**Nutrition status (n = 212)**						
Severely underweight	Reference					
Underweight	0.97	0.38–2.47	0.956			
Normal weight	1.05	0.50–2.23	0.892			
**Outcome at discharge (n = 263)**				–	–	–
Alive	Reference					
Died	0.54	0.12–2.50	0.431			
**Binary Treatment outcomes** ^b^ **(n = 263)**				–	–	–
Unfavourable	Reference					
Favourable	0.85	0.44–1.64	0.627			

^a^ For each outcome, 1 April 2020 was the cut-off date dividing TBM episodes before and during the COVID-19 pandemic and we included all the data in this analysis. We therefore compared 1 January 2017–31 March 2020 (pre-pandemic) to 1 April 2020–31 December 2021 (during the pandemic). ^b^ Favourable outcomes referred to cure or treatment completion whereas unfavourable outcomes referred to death, loss to follow-up, or treatment failure. BCG: *bacille Calmette–Guérin*, HIV: human immunodeficiency virus, TPT: TB preventive treatment, OR: odds ratio, aOR: adjusted odds ratio.

## Data Availability

Data can be made available upon reasonable request.

## Supplementary Materials

The following supporting information can be downloaded at: https://www.mdpi.com/article/10.3390/tropicalmed10050127/s1, Table S1: Xpert MTB/RIF and culture for *Mycobacterium tuberculosis* results for specimen other than cerebrospinal fluid for children with tuberculous meningitis at Tygerberg hospital (n = 263); Table S2: Microbiologically confirmed *Mycobacterium tuberculosis* disease by cerebrospinal fluid specimen for children with tuberculous meningitis at Tygerberg hospital (n = 263); Table S3: Microbiologically confirmed *Mycobacterium tuberculosis* disease by specimen other than cerebrospinal fluid for children with tuberculous meningitis at Tygerberg hospital (n = 263):, Table S4: Microbiologically confirmed *Mycobacterium tuberculosis* disease by cerebrospinal fluid and/or other specimen in children with tuberculous meningitis at Tygerberg Hospital between 2017–2021 (n = 263).
