# Supplementary material for: Incidence, Disease Spectrum, and Outcomes of Tuberculous Meningitis in South African Children: The Initial Impact of COVID-19
Source: Trop Med Infect Dis. 2025 May 7;10(5):127. doi: 10.3390/tropicalmed10050127 (PMC12115567; doi:10.3390/tropicalmed10050127)
Supplement: Supplementary file 1 [file tropicalmed-10-00127-s001.zip › tropicalmed-3583601-supplementary.pdf]

Supplementary Materials: S1, S2, S3 and S4.

Table S1. Xpert MTB/RIF and culture for *Mycobacterium tuberculosis* results for specimen other than cerebrospinal fluid for children with tuberculous meningitis at Tygerberg hospital (n=263)

	**Prior to COVID-19 pandemic**	**During COVID-19 pandemic**	**Total**	**p-value**
	n (%)	n (%)	N (%)	
**Number (%)**	198 (75.3)	65 (24.7)	263 (100.0)	
**Other specimen Xpert[a] (n=207)**				
Positive	84 (55.3)	23 (41.8)	107 (51.7)	0.087
Negative	68 (44.7)	32 (58.2)	100 (48.3)	
**Other specimen culture[b] (n=186)**				
Positive	73 (53.7)	19 (38.0)	92 (49.5)	0.058
Negative	63 (46.3)	31 (62.0)	94 (50.5)	

[a]Fifty-six children had unknown or missing other specimen CSF Xpert MTB/RIF results
[b]Seventy-seven children had unknown or missing other specimen *M.tb* culture results

Table S2. Microbiologically confirmed *Mycobacterium tuberculosis* disease by cerebrospinal fluid specimen for children with tuberculous meningitis at Tygerberg hospital (n=263)

	**Prior to COVID-19 pandemic**	**During COVID-19 pandemic**	**Total**	**p-value**
	n (%)	n (%)	N (%)	
**Number (%)**	198 (75.3)	65 (24.7)	263 (100.0)	
**CSF Microbiology results[a]**				
Xpert and/or culture positive	63 (31.8)	26 (40.0)	89 (33.8)	0.197
Xpert and/or culture negative	88 (44.4)	30 (46.2)	118 (44.9)	
Xpert and/or culture unknown or missing	47 (23.7)	9 (13.8)	56 (21.3)	

[a]Fifty children had a positive CSF Xpert MTB/RIF. Eleven children had a positive culture for *M.tb*. Four children had both CSF Xpert MTB/RIF and *M.tb* culture results as positive. Three children had Xpert MTB/RIF trace positive and *M.tb* culture positive results.
CSF- cerebrospinal fluid

Table S3. Microbiologically confirmed *Mycobacterium tuberculosis* disease by specimen other than cerebrospinal fluid for children with tuberculous meningitis at Tygerberg hospital (n=263)

	**Prior to COVID-19 pandemic**	**During COVID-19 pandemic**	**Total**	**p-value**
	n (%)	n (%)	N (%)	
**Number (%)**	198 (75.3)	65 (24.7)	263 (100.0)	
**Other specimen Microbiology results[a]**				
Xpert and/or culture positive	100 (50.5)	26 (40.0)	126 (47.9)	0.043
Xpert and/or culture negative	61 (30.8)	31 (47.7)	92 (35.0)	

Xpert and/or culture unknown or missing	37 (18.7)	8 (12.3)	45 (17.1)

[a]Of 89 children with a positive CSF Xpert MTB/RIF and/or *M.tb* culture, 50 children also had positive Xpert MTB/RIF and/or *M.tb* culture from specimen other than CSF. Of 118 children who had a negative CSF Xpert MTB/RIF and/or *M.tb* culture, 45 had positive Xpert MTB/RIF and/or *M.tb* culture from specimen other than CSF. Of 56 children who had unknown or missing CSF Xpert MTB/RIF and *M.tb* culture results, 27 children had positive Xpert MTB/RIF and/or *M.tb* culture from specimen other than CSF.

CSF- cerebrospinal fluid

Table S4. Microbiologically confirmed *Mycobacterium tuberculosis* disease by cerebrospinal fluid and/or other specimen in children with tuberculous meningitis at Tygerberg Hospital between 2017-2021 (n=263)

	**Other specimen Xpert and/or culture**			
**CSF Xpert and/or culture**	**Positive**	**Negative**	**Unknown**	**Total**
Positive	50	20	19	89
Negative	49	51	18	118
Unknown	27	21	8	56
**Total**	126	92	45	263

Of 174 children with a negative or unknown CSF Xpert MTB/RIF and/or culture for M.tb, 76 children had a positive Xpert MTB/RIF and/or *M.tb* culture by specimen other than CSF.

CSF- cerebrospinal fluid
